# The Metabolic Signatures Associated with the Effects of Aerobic Exercise on Depressive-like Behaviors in CUMS Rats

**DOI:** 10.3390/metabo16020114

**Published:** 2026-02-05

**Authors:** Huan Xiang, Danhui Zhang, Yuchen Zhu, Jiangtao Hou, Yumei Han

**Affiliations:** 1 School of Physical Education, Shanxi University, Taiyuan 030006, China; xianghuan0327@sxu.edu.cn (H.X.); zhangdanhui@sxu.edu.cn (D.Z.); zhuyuchen1@sxu.edu.cn (Y.Z.); 2 Department of Physical Education, Changzhi Preschool Education College, Changzhi 046000, China

**Keywords:** CUMS, aerobic exercise, LC-MS, ^1^H-NMR

## Abstract

**Objectives**: This study explored the antidepressant mechanisms of aerobic exercise in CUMS rats by analyzing urinary metabolomics (LC-MS and NMR), with the aim of providing both theoretical and practical support for exercise-based depression interventions. **Methods**: (1) Thirty-two Sprague-Dawley rats were acclimatized for one week and then randomly assigned to four groups (n = 8 per group): control (C), control + aerobic exercise group (E), CUMS model (D), and CUMS + exercise (DE). Groups D and DE were subjected to nine types of CUMS stimuli. Behavioral indicators were assessed weekly, and the successful establishment of the CUMS model was confirmed at week 3. Following successful modeling, rats in groups E and DE underwent four weeks of aerobic exercise training. Throughout this period, groups D and DE continued to receive CUMS exposure, while groups C and E were maintained under standard control conditions. (2) At the end of week 7, behavioral tests were repeated. Twelve-hour urine samples were collected for metabolomic analysis using liquid chromatography–mass spectrometry (LC-MS) and ^1^H-NMR spectroscopy. The following morning, rats were euthanized under anesthesia. Whole blood was collected from the abdominal aorta, and serum was separated for subsequent biochemical assays. Bioinformatics approaches were employed to identify potential targets and signaling pathways associated with the antidepressant effects of aerobic exercise. (3) For statistical analysis, one-way or two-way analysis of variance (ANOVA) was applied to behavioral, physiological, and biochemical data, whereas multivariate statistical analysis was used for metabolomic data. **Results**: (1) By week 3, body mass, sucrose preference, rearing frequency, and the number of grid crossings were significantly lower in groups D and DE than in groups C and E (*p* < 0.05 or *p* < 0.01). These findings confirmed the successful establishment of the depression model. At week 7, all behavioral indicators in group DE showed significant recovery relative to group D (*p* < 0.05 or *p* < 0.01). (2) Compared with group C, corticosterone and blood ammonia levels were significantly elevated in group D (*p* < 0.01). In contrast, these levels were markedly reduced in group DE compared with group D (*p* < 0.01). (3) LC-MS analysis identified 25 urinary metabolites associated with depression in group D relative to group C. Among these, 21 were significantly downregulated and 4 were upregulated (*p* < 0.05 or *p* < 0.01), involving seven metabolic pathways. Following aerobic exercise intervention, six of these depression-related metabolites in group DE showed significant recovery (*p* < 0.05 or *p* < 0.01), which were associated with two metabolic pathways. (4) Integrated analysis of LC-MS and ^1^H-NMR data revealed glutamine as a common differential metabolite, linked to three metabolic pathways. All metabolic pathways modulated by aerobic exercise were related to amino acid metabolism. (5) Bioinformatics analysis indicated that AKT1, MTOR, IL6, RAF1, and TNF were core targets through which aerobic exercise regulated urinary metabolism in CUMS rats. **Conclusions**: A four-week regimen of aerobic exercise significantly improved depressive-like behaviors and enhanced anti-fatigue capacity in CUMS rats. This exercise regimen promoted urinary metabolic remodeling, primarily through the modulation of amino acid metabolism. Furthermore, its antidepressant effect is likely mediated through the regulation of core tissue targets—including AKT1, mTOR, IL-6, RAF1, and TNF—thereby influencing key pathways such as PI3K-AKT, MAPK/ERK, and neuroinflammatory signaling.

## 1. Introduction

Depression is a prevalent mental disorder characterized by high rates of incidence, disability, and recurrence. It has emerged as a leading cause of global disease burden, affecting approximately 300 million people worldwide [1]. The etiology of depression is complex, involving a combination of genetic, psychological, and biochemical factors. Multiple pathological mechanisms have been implicated in depression, primarily including the monoamine hypothesis, hypothalamic–pituitary–adrenal (HPA) axis dysregulation, and neuroinflammation/cytokine hypotheses [2]. While these theories provide valuable insights, they largely address the pathogenesis from isolated perspectives, lacking a comprehensive explanation for the systemic metabolic alterations observed in depressed individuals or animal models.

Exercise therapy, recognized as a cost-effective, safe, and easily implementable intervention, has been endorsed by global experts as a key strategy for mitigating the onset and progression of depression [3]. Aerobic exercise, a continuous mode of physical activity primarily fueled by the aerobic metabolic system, has been shown to exert more potent regulatory effects on the brain compared to other exercise forms. Specifically, sustained aerobic exercise enhances cardiopulmonary and immune function, improves psychological well-being, and modulates cerebral glucose metabolism [4]. Studies have demonstrated that aerobic exercise alleviates depression through various mechanisms, including the suppression of inflammatory responses, regulation of neurotransmitter expression, reduction in hippocampal neuron apoptosis, promotion of neurogenesis, and positive modulation of HPA axis function [5,6]. However, research on how aerobic exercise modulates the systemic metabolic profile in individuals with depression remains relatively limited.

Metabolomics is an omics technology dedicated to the comprehensive analysis of metabolic profiles within biological systems. This approach allows for the precise detection of alterations in endogenous metabolites in response to internal perturbations and has been extensively applied in fields such as infectious disease control, pharmaceutical development, and mechanistic studies of diseases [7]. Commonly employed analytical techniques in metabolomics include nuclear magnetic resonance spectroscopy (NMR), gas chromatography–mass spectrometry (GC-MS), and liquid chromatography–mass spectrometry (LC-MS). NMR offers advantages such as non-destructiveness, minimal sample preparation, rapid analysis, and unbiased detection but is limited by relatively low sensitivity and a constrained range of detectable compounds [8]. In contrast, LC-MS provides high sensitivity, fast analysis, and strong separation capabilities, although its incomplete database can pose challenges for compound identification [9]. GC-MS is primarily suited for volatile compounds and benefits from a well-established database, which simplifies identification [10]. To date, few studies have leveraged the complementary strengths of multiple metabolomic platforms for cross-validation to investigate the mechanisms underlying exercise interventions for depression.

Multiple sample types are suitable for metabolomic analysis. Blood is tightly regulated by homeostatic mechanisms; significant and stable changes in the blood metabolome typically occur only when homeostasis is disrupted and are often disease-associated. Consequently, blood-based omics studies are valuable for disease diagnosis [11]. Urine, on the other hand, is a major reservoir of metabolic waste. Unlike blood, its composition is not under strict homeostatic control, allowing the urine proteome and metabolome to exhibit changes even in early disease stages. Moreover, urine collection is non-invasive, simple, and convenient while yielding rich biomarker information, which accounts for its broad application potential in clinical diagnosis and health monitoring [12].

This study investigated the interventional effects of aerobic exercise on rats with depressive-like behaviors induced by chronic unpredictable mild stress (CUMS). Building on our research group’s previous metabolomic analysis of blood samples using NMR and LC-MS techniques [13], the current work further deepens the understanding of the antidepressant effects of aerobic exercise by profiling the metabolites of end products in the urine of depressed rats. Additionally, integrated bioinformatics systems analysis was employed to identify the therapeutic targets and signaling pathways through which aerobic exercise intervenes in depression, aiming to comprehensively reveal the metabolic mechanisms underlying the antidepressant effect of aerobic exercise.

Based on this, we propose the following scientific hypothesis: Aerobic exercise can improve depressive-like behaviors and anti-fatigue capacity in CUMS rats by regulating urinary metabolic remodeling. Furthermore, this peripheral urinary metabolic remodeling is associated with amino acid metabolism in the central hippocampal region and pathways mediated by core targets, collectively constituting the key metabolic mechanism underlying the antidepressant effect of aerobic exercise.

## 2. Materials and Methods

### 2.1. Instruments and Reagents

The main experimental instruments included: an ultra-high performance liquid chromatography–mass spectrometer (UltiMate 3000 UHPLC Q Exactive Orbitrap-MS, Thermo Fisher Scientific Inc., Waltham, MA, USA), an ACQUITY UPLC HSS T3 column (2.1 mm × 100 mm, 1.8 μm, Waters Corporation, Milford, MA, USA), a Neofuge 13R high-speed desktop refrigerated centrifuge (Shanghai Lishen Scientific Instruments Co., Ltd., Shanghai, China), and a vacuum freeze dryer (Shanghai Zhixin Instrument Technology Co., Ltd., Shanghai, China). The main reagents were chromatographically pure acetonitrile and pure formic acid (Thermo Fisher Scientific Inc., Waltham, MA, USA).

### 2.2. Experimental Animals and Grouping

**Figure 1:**
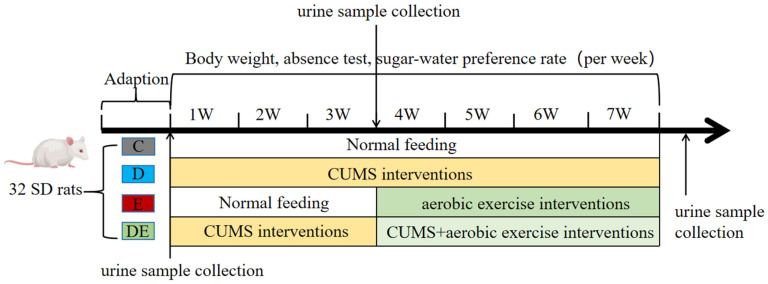
Design and timeline of animal experiment.

Eight-week-old male Sprague-Dawley (SD) rats were purchased from Beijing Weitong Lihua Experimental Animal Technology Co., Ltd. (Beijing, China) (Animal License No.: SCXK (Jing) 2021-0006). The rats were raised in an environment with a temperature of (25 ± 2) °C, a humidity of (50 ± 20)%, and a 12-h light-dark cycle. After 1 week of adaptive feeding, the rats were randomly divided into four groups: the control group (Group C), the control + aerobic exercise group (Group E), the CUMS model group (Group D), and the model + exercise group (Group DE). Using G*Power 3.1 software, a two-way mixed analysis of variance (ANOVA) was employed for the hypothesis test (power = 0.8, α = 0.05, within-between interaction, effect size = 0.4). The calculation results indicated that 8 rats per group are required to meet the statistical requirements. Each group contained 8 rats with a total of 32. The rats were randomly assigned to the four experimental groups using a random number table method, to ensure that baseline differences among groups were minimized. The rats were single caged (cage size: length 420 mm, width 240 mm and height 240 mm). Groups D and DE were subjected to 7 weeks of CUMS modeling, while Groups E and DE received aerobic exercise starting from the 4th week. The experimental groups and timelines are shown in Figure 1. This experimental protocol was approved by the Scientific Research Ethics Committee of Shanxi University (Approval No.: SXULL2025062), and the experiment was conducted in accordance with the guidelines for animal care and use.

### 2.3. Aerobic Exercise Protocol

From the fourth week with CUMS, rats in group A exercised on a motor-driven treadmill for 30 min, five times per week for four weeks. The treadmill speed started from 3 m/min for 5 min, increased to 5 m/min for 5 min, then to 8 m/min for 20 min. To diminish the stress from unfamiliar activities, rats were familiarized with the treadmill for three consecutive days (15 min per day) before the commencement of formal exercise intervention. They ran at the speed of 3 m/min for the first 5 min, 5 m/min for the second 5 min and 8 m/min for the last 5 min (Table 1).

### 2.4. Replication of CUMS Depression Model

**Figure 2:**
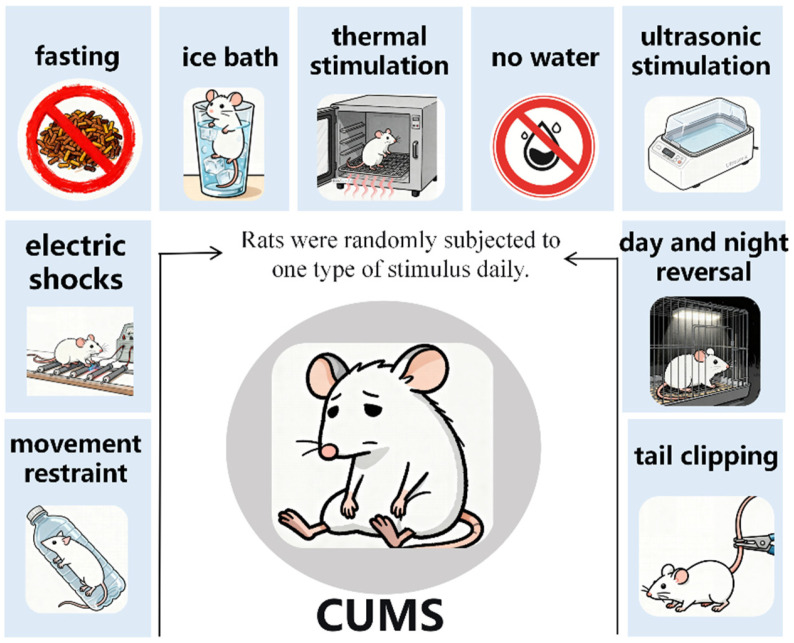
CUMS modeling protocol.

Rats in Groups D (CUMS model group) and DE (model + exercise group) were randomly subjected to one type of stimulus per day during the modeling period. The following modeling stimuli were applied: no water for 24 h, 4 °C ice bath for 5 min, fasting for 24 h, thermal stimulation for 10 min, ultrasonic stimulation for 3 h, and electric shocks to feet (voltage 36 V, shock interval 10 s, for 10 times), movement restraint for 3 h, tail clipping for 2 min, day and night reversal (placed under fluorescent light for 24 h). This modeling process lasted for 7 weeks, while groups C and E were raised under normal control conditions. Conduct behavioral tests every weekend, and subsequent experiments were carried out after the successful establishment of the model was confirmed at the end of the 3rd weekend. The replication process of the CUMS model and the aerobic exercise protocol referred to the mature methods previously established by the research group [14]. The specific procedure is shown in Figure 2.

### 2.5. Behavioral Tests

All behavioral tests were conducted in a quiet, temperature-controlled room (22 ± 1 °C, 50 ± 5% relative humidity) between 09:00 and 12:00 a.m. to minimize the confounding effects of circadian rhythms and environmental disturbances. The testing arena was thoroughly cleaned with 75% ethanol and dried before each trial to eliminate odor cues from previous rats. Behavioral tests included the sucrose preference test (SPT) and the open field test (OFT). The detailed test procedure has been presented in the previous report of the research group [13]. All behavioral tests were conducted by researchers who were blinded to the group assignments of the rats, to avoid subjective bias. All behavioral test videos were scored twice by the same trained researcher at an interval of two weeks. The intraclass correlation coefficient (ICC) was calculated to verify consistency; the results showed ICC > 0.90, indicating excellent intra-observer reliability.

### 2.6. Sample Collection and Pretreatment

Two hours after the last aerobic exercise session, all rats underwent behavioral tests. Following the tests, all rats were fasted overnight with free access to water only. The next morning, after the rats were anesthetized, blood samples were collected via the abdominal aorta for the determination of blood biochemical indicators. Urine samples from each group of rats were collected on ice using metabolic cages for 12 h. The collected urine samples were centrifuged at 13,000 rpm for 20 min, and the supernatant was taken as the sample to be tested.

### 2.7. Determination of Blood Biochemical Indicators

The physiological and biochemical indicators included testosterone, corticosterone, blood ammonia, hemoglobin, and lactic acid. All these indicators were determined in accordance with the instructions provided with the test kits. The test kits were purchased from Nanjing Jiancheng Bioengineering Institute, Nanjing, China.

### 2.8. Urine Metabolomics Detection

#### 2.8.1. LC-MS Metabolomics Detection

##### Sample Pretreatment

After thawing, urine samples were centrifuged at a high speed of 13,000 rpm for 10 min to remove solid substances. The supernatant was diluted with distilled water, methanol, and acetonitrile at a ratio of 1:1:1, vortex-mixed, and then filtered through a 0.22 μm membrane filter respectively. The optimal sample pretreatment method was selected based on the number of detected chromatographic peaks, resolution, and intensity. Quality Control (QC) samples were prepared by mixing 10 μL of each urine sample to be tested, following the same sample pretreatment procedure.

##### LC-MS Analysis Conditions

Liquid Chromatography Conditions: A Waters ACQUITY UPLC HSS T3 column was used for detection. Mobile phase A was water containing 0.1% formic acid, and mobile phase B was acetonitrile containing 0.1% formic acid. Different elution gradients were applied, with a flow rate of 0.2 mL/min, an injection volume of 5 μL, and a column temperature of 40 °C.

Mass Spectrometry Conditions: Electrospray ionization (ESI) was adopted, with a positive-negative ion switching acquisition mode and a Full Scan/dd-MS2 scanning mode. The spray voltage was 3.5 kV for the positive ion mode and 2.5 kV for the negative ion mode. The capillary temperature was 320 °C, and the heater temperature was 300 °C. The sheath gas flow rate was 35 arb, and the auxiliary gas flow rate was 10 arb. The resolution was set to 35,000 FWHM for MS Full Scan and 17,500 FWHM for MS/MS, while the normalized collision energy (NCE) was set to 12.5 eV, 25 eV, and 37.5 eV. The m/z acquisition range was 100–1500 Da.

Data Processing: The liquid chromatography–mass spectrometry spectra and data of urine samples from each group were imported into Compound Discoverer 3.1 software (Thermo Fisher Scientific Inc., Waltham, MA, USA) for pretreatment to obtain matched and aligned peak data. The parameters were set as follows: mass range of 100–1500 Da, mass deviation of 5 ppm, retention time tolerance of 0.05, and signal-to-noise ratio threshold of 1.5. The obtained peak data were imported into Excel for peak area normalization, and then into SIMCA-P 14.1 software (Umetrics, Umeå, Sweden) for principal component analysis (PCA), partial least squares discriminant analysis (PLS-DA), and orthogonal partial least squares discriminant analysis (OPLS-DA). Finally, differential metabolites with significant changes among groups were identified by screening the intersection of variables with variable importance in projection (VIP) > 1 in the S-plot, those with *p* < 0.05 in the independent sample *t*-test, and those with FDR-adjusted q < 0.10. These differential metabolites were identified using online databases such as the Human Metabolome Database (HMDB) and Kyoto Encyclopedia of Genes and Genomes (KEGG). The data of the identified differential metabolites were imported into MetaboAnalyst 3.0 for pathway enrichment analysis to explore the metabolic pathways involved in the differential metabolites.

#### 2.8.2. ^1^H-NMR Metabolomics Detection

Urine sample pretreatment: Preparation of the samples for metabolomic analysis by ^1^H-NMRwas performed as described previously. 500 μL of urine sample was transferred to an Eppendorf tube after thawing, and 200 μL phosphate-buffered solution (0.2 mol/L Na_2_HPO_4_, 0.2 mol/LNaH_2_PO_4_, pH = 7.40) containing D_2_O was added. Then an appropriate amount of 0.15% sodium 3-trimethylsilyl-(2,2,3,3-d4)-1-propionate (TSP) was added as a calibration of the zero point of chemical shift. The sample was then centrifuged at 13,000 rpm for 20 min at 4 °C. 600 μL of the supernatant was placed in a nuclear magnetic tube with an inner diameter of 5 mm.

NMR detection conditions: The ^1^H-NMR spectra of the urine samples were obtained using a Bruker 600 MHz AVANCE III NMRspectrometer (Bruker Biospin, Rheinstetten, Germany) operating at a ^1^H frequency of 600.13 MHz and a temperature of 298 K. Samples were analyzed using the NOESY pulse sequence. The parameters were set to a spectral width of 8 kHz, a mixing time of 150 ms, a relaxation delay of 320 ms, sampling point of 64 k, and accumulation number of 64. The pre-saturation method was used to suppress the water peak during the relaxation delay, the spectrometer bias is set at the water peak position, and the free induction decay signal was converted into a ^1^H-NMR spectrum by Fourier transformation.

NMRspectrum processing: Urine ^1^H-NMR spectra were adjusted for baseline and phase distortions using the MestReNova software (ver. 8.0.1, Mestrelab Research, Santiago de Compostela, Spain). The chemical shift correction of the NMR spectra was performed based on the chemical shift (δ0.0) of TSP. With δ0.01 as the basic unit, the integral of the region of δ0.8~8.0 in the spectrum could be obtained, and the integral value corresponding to the chemical displacement value segment was acquired. In order to eliminate the influence of water peak and urea peak on the analysis results, the peak intensity of the δ4.6~6.2 interval was set to zero. Then, the normalization method was used to eliminate the influence of the difference in sample concentration, so that the data was limited to the range of δ0.0~1.0. After data normalization, multivariate statistical analysis was performed.

### 2.9. Bioinformatics Analysis of Aerobic Exercise in Depression Intervention

#### 2.9.1. Prediction of Gene Targets Related to Aerobic Exercise and Depression

Based on the Genecards database (https://www.genecards.org/, (accessed on 22 August 2025)), the keywords “Aerobic exercise” and “Depression” were entered separately for retrieval. After exporting the retrieved data of potential target genes related to aerobic exercise and depression, the data were input into Venny 2.1 (https://bioinfogp.cnb.csic.es/tools/venny/, (accessed on 22 August 2025)) to screen and obtain the overlapping targets of the two. These overlapping targets were used to determine the potential target genes of aerobic exercise in intervening depression.

#### 2.9.2. Construction of PPI Network

The screened potential target genes were imported into the STRING 12.0 platform (with the species set as Rattus norvegicus and the Minimum required interaction score set as 0.4) to construct a Protein-Protein Interaction (PPI) network. Cytoscape 3.7.2 software was used for visual analysis of the constructed PPI network.

#### 2.9.3. GO and KEGG Enrichment Analyses

The DAVID database (https://davidbioinformatics.nih.gov/, (accessed on 22 August 2025)) was used to perform bioinformatics enrichment analysis on the aforementioned overlapping targets. According to the analysis results, significant results with *p* < 0.05 were screened. The top 10 Gene Ontology (GO) terms and top 20 Kyoto Encyclopedia of Genes and Genomes (KEGG) pathway terms with the smallest *p*-values were visualized. This was done to further explore the potential roles of these target genes in biological processes (BP), cellular components (CC), molecular functions (MF), and signaling pathways.

#### 2.9.4. Construction of Target-Pathway Network and Screening of Core Targets

The obtained core targets were matched with KEGG pathway data, and Cytoscape 3.7.2 software was used to construct a multi-dimensional “target-pathway” network. Topological analysis was then conducted on the network. Key active components, key targets, and key pathways were screened based on the criteria of degree, betweenness, and closeness.

### 2.10. Statistical Analysis

IBM SPSS Statistics 26 and GraphPad Prism 10.1 software were used for statistical analysis of data and graphing. For the data collected in the 3rd week, one-way analysis of variance (one-way ANOVA) was applied. Multiple comparisons between groups were conducted using the least significant difference (LSD) test, while the Kruskall–Wallis test was used for data that did not conform to a normal distribution. For the data collected in the 7th week, after testing for normal distribution and homogeneity of variance, two-way analysis of variance (two-way ANOVA) was used, followed by Bonferroni post -hoc test. All data were expressed as mean ± standard deviation (mean ± SD), and a *p*-value < 0.05 was considered statistically significant.

## 3. Results

### 3.1. Four Weeks of Aerobic Exercise Improved Depressive-like Behaviors in CUMS Rats

**Figure 3:**
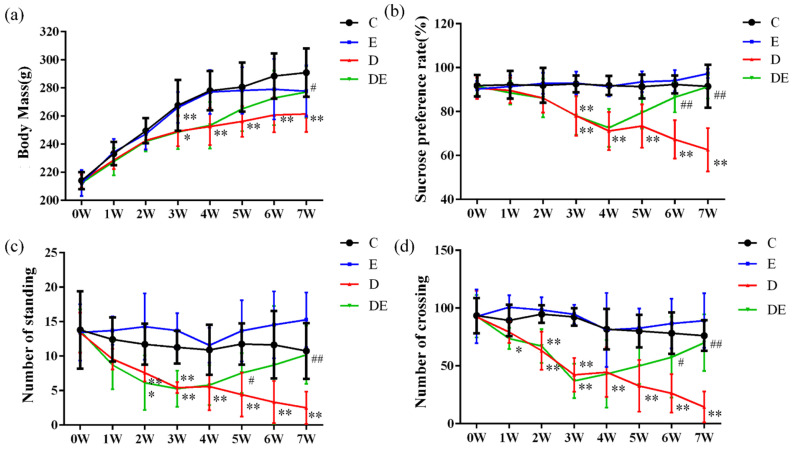
Analysis of behavioral indexes of rats. Body mass and behavioral test results of rats in each group at week 0 to week 7. (**a**) Body mass; (**b**) Sucrose preference rate; (**c**) Number of standing; (**d**) Number of crossing. * *p* < 0.05, ** *p* < 0.01 versus Group C; ^#^ *p* <0.05, ^##^ *p* < 0.01 versus Group D.

The sucrose preference test (SPT) and open field test (OFT) were mainly used to assess whether the animals exhibited anhedonia, as well as their spontaneous activity and exploratory behavior. As shown in Figure 3, in the 3rd week, the body weight (BW), sucrose preference rate, rearing frequency, and grid crossing number of rats in Group D (CUMS model group) and Group DE (model + exercise group) were significantly lower than those in Group C (control group) and Group E (control + aerobic exercise group) (*p* < 0.05 or *p* < 0.01). This indicated that the rat depression model was successfully established. In the 7th week, compared with Group D, all behavioral indicators of rats in Group DE showed a significant recovery (*p* < 0.05 or *p* < 0.01). The above results suggested that four weeks of aerobic exercise could effectively improve the depressive-like behaviors in CUMS rats.

### 3.2. Four Weeks of Aerobic Exercise Enhanced Anti-Fatigue Ability in CUMS Rats

Depression is mainly characterized by symptoms such as low mood, loss of interest, insufficient energy, and easy fatigue [15]. Fatigue is a very common symptom among patients with depression. Some biochemical indicators in the blood—such as blood testosterone, blood ammonia, hemoglobin, and blood lactic acid—are often used to reflect the body’s anti-fatigue ability. Additionally, adrenocortical hormones (primarily corticosterone) in rodents are closely related to the occurrence and development of depression [16]. The results of this study (Table 2) showed that compared with Group C (control group), the levels of corticosterone and blood ammonia in Group D (CUMS model group) were significantly increased (*p* < 0.01), suggesting that CUMS may induce fatigue symptoms in rats. In contrast, compared with Group D, the levels of corticosterone and blood ammonia in Group DE (model + exercise group) were significantly decreased (*p* < 0.01), indicating that aerobic exercise can effectively improve fatigue symptoms in CUMS rats.

### 3.3. Four Weeks of Aerobic Exercise Remodeled the Urinary Amino Acid Metabolic Profile of CUMS Rats

#### 3.3.1. Results of LC-MS Metabolomics Detection

- Multivariate Statistical Analysis

**Figure 4:**
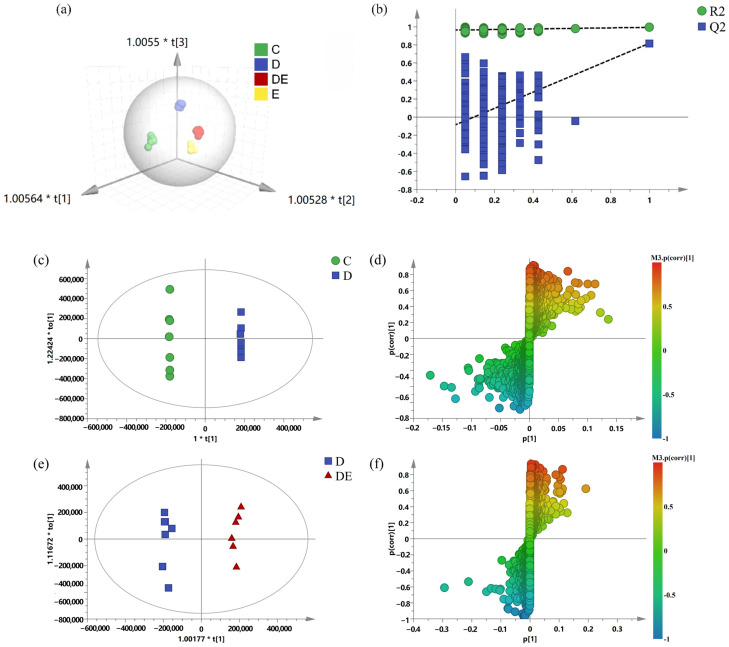
Multivariate statistical analysis of LC-MS untargeted metabolomics. Metabolomics multivariate statistical analyses were performed to test the reliability of metabolomics assay parameters as well as urinemetabolic profiles in each group of rat urine (of which the QC group is a sample used for quality control) (n = 6). (**a**) 3D plots of OPLS-DA scores for each group; (**b**) PLS-DA model validation plots; The horizontal dashed line corresponding to R_2_ represents the original R^2^ value of the model, and the slanted dashed line corresponding to Q_2_ represents the trend line of Q_2_ values as the number of permutations increases. (**c**) plots of OPLS-DA scores for group C vs. group D; (**d**) S-Plot plots for group C vs. group D; (**e**) plots of OPLS-DA scores for group D vs. group DE; (**f**) S-Plot plots for group D vs. group DE. The “*” here is a multiplication symbol, and the preceding numbers (e.g., 1.00564, 1.00528, 1.0055) are the scaling factors for the corresponding principal components.

Orthogonal partial least squares discriminant analysis (orthogonal PLS-DA, OPLS-DA) is a supervised multivariate statistical analysis method. It can effectively eliminate irrelevant influences on the study and help understand the overall metabolic differences among samples of different groups as well as the differences between groups. The OPLS-DA results showed that the metabolic profiles of each group exhibited a clustering trend, with a significant separation trend between groups. Group C (control group) and Group D (CUMS model group) were clearly separated, indicating that CUMS modeling had a significant impact on the urinary metabolic profile of rats. Additionally, the metabolic profiles of Group DE (model + exercise group) and Group D were also clearly separated, suggesting that aerobic exercise had a significant regulatory effect on the urinary metabolism of CUMS rats (see Figure 4c,e). Moreover, the QC samples showed good clustering, which indicated that the analytical method was stable and reliable and could meet the analytical requirements for large-batch sample injection (see Figure 4a). Partial least squares discriminant analysis (PLS-DA) was used to perform a permutation test on the above model data. It was found that the regression line of Q^2^ had an intercept less than 0 on the ordinate, indicating that there was no overfitting phenomenon (see Figure 4b).

b. Differential Metabolites Analysis

The screening criteria for differential metabolites among groups were as follows: VIP > 1, *p* < 0.05, and FDR-adjusted q < 0.10 (see Figure 4d,f). A total of 25 metabolites related to depression were identified. Comparison of metabolite contents showed the following: Compared with Group C (control group), the levels of 21 metabolites in the urine of rats in Group D (CUMS model group) were significantly decreased, including phenylacetylglycine, 3-indoxyl sulfate, and 7-methylguanine. In contrast, the levels of 4 metabolites (caprolactam, cholic acid, and tyramine) were significantly increased in Group D. After 4 weeks of aerobic exercise, 6 differential metabolites in Group DE (model + exercise group) were significantly altered. Compared with Group D, the contents of metabolites including 3-indoxyl sulfate, 3-hydroxyvaleric acid, and methylthioadenosine in Group DE were significantly increased, while the levels of caprolactam, cholic acid, and tyramine were significantly decreased (see Table 3).

c. Metabolic Pathway Analysis

**Figure 5:**
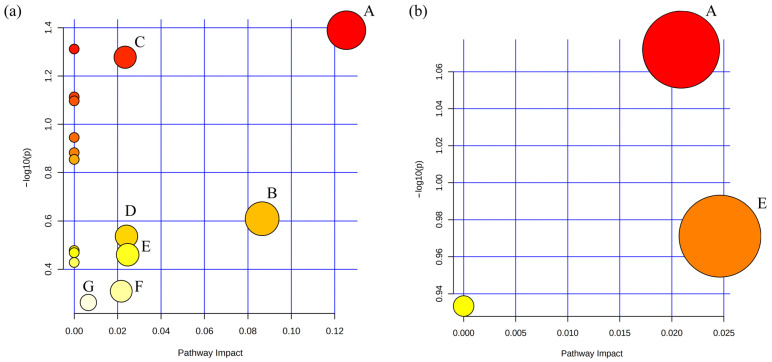
LC-MS untargeted metabolomics differential metabolite and metabolic pathway analysis. Metabolic pathway analysis was performed using Metabo Analyst 5.0 to compare the differential metabolites between groups C and D, between groups D and DE. (**a**) Depression-related metabolic pathways; (**b**) Aerobic exercise modulates depression-related metabolic pathways. A: cysteine and methionine metabolism; B: alanine, aspartate, and glutamate metabolism; C: arginine and proline metabolism; D: glycine, serine, and threonine metabolism; E: tyrosine metabolism; F: purine metabolism; G: steroid hormone biosynthesis.

Metabo Analyst 5.0 was used to perform metabolic pathway analysis on the aforementioned differential metabolites. A metabolic pathway with an impact value > 0.1 was considered the pathway with the highest contribution. The results showed that a total of 7 metabolic pathways related to depression were identified, as shown in Figure 5a. These pathways were: cysteine and methionine metabolism; alanine, aspartate and glutamate metabolism; arginine and proline metabolism; glycine, serine and threonine metabolism; tyrosine metabolism; purine metabolism; and steroid hormone biosynthesis. Two metabolic pathways were involved in the regulatory effect of exercise, as shown in Figure 5b. These two pathways were cysteine and methionine metabolism, and tyrosine metabolism.

#### 3.3.2. Comparative Analysis of LC-MS and ^1^H-NMR Urine Metabolomics

- Comparative Analysis of Depression-Related Differential Metabolites and Metabolic Pathways

The results of detecting and analyzing depression-related differential metabolites and their metabolic pathways in the urine of CUMS rats using LC-MS and ^1^H-NMR metabolomics techniques are shown in Table 4. The ^1^H-NMR urine metabolomics results were derived from the previous work of this research group [14].

**Figure 6:**
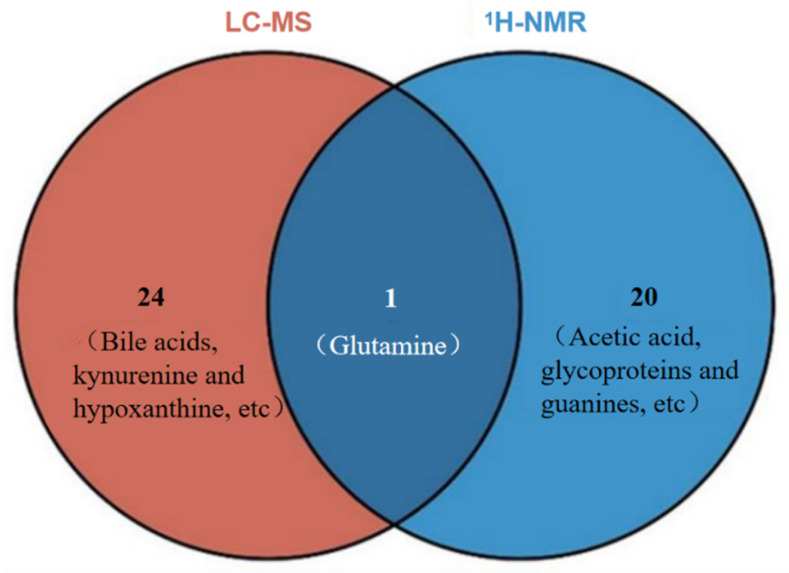
LC-MS untargeted metabolomics differential metabolite and metabolic pathway analysis.

The ^1^H-NMR technique detected a total of 21 depression-related metabolites in the urine of CUMS rats, including acetic acid, glycoproteins, and glutamine. These metabolites were involved in 8 metabolic pathways, such as alanine, aspartate and glutamate metabolism, tricarboxylic acid (TCA) cycle, and butyrate metabolism. In contrast, the LC-MS technique detected 25 depression-related metabolites in the urine of CUMS rats, including phenylacetylglycine, 3-indoxyl sulfate, and 7-methylguanine. These metabolites were involved in 7 metabolic pathways, such as cysteine and methionine metabolism, alanine, aspartate and glutamate metabolism, and arginine and proline metabolism. Glutamine was the only shared depression-related metabolite between the two metabolomics techniques (Figure 6). There were 3 shared depression-related metabolic pathways, namely “arginine and proline metabolism”, “glycine, serine and threonine metabolism”, and “alanine, aspartate and glutamate metabolism”—all of which belong to amino acid metabolism.

b. Comparative Analysis of Aerobic Exercise-Related Antidepressant Metabolites and Metabolic Pathways

The results of analyzing the depression-related differential metabolites in CUMS rats’ urine regulated by aerobic exercise, based on LC-MS and ^1^H-NMR metabolomics techniques, are shown in Table 5. The ^1^H-NMR technique detected 5 metabolites significantly regulated by 4 weeks of aerobic exercise, including glutamine, acetone, and pyruvate. These metabolites were involved in 5 metabolic pathways, such as alanine, aspartate and glutamate metabolism, tricarboxylic acid (TCA) cycle, and glycolysis or gluconeogenesis. On the other hand, the LC-MS technique detected 6 metabolites significantly regulated by aerobic exercise, including 3-indoxyl sulfate, caprolactam, and cholic acid. These metabolites were involved in 2 metabolic pathways: cysteine and methionine metabolism, and tyrosine metabolism. Although there were no identical metabolites or metabolic pathways between the detection results of ^1^H-NMR and LC-MS, both sets of metabolic pathways involved amino acid metabolism. This suggests that amino acid metabolism may be one of the important pathways for exercise-induced antidepressant effects.

### 3.4. Bioinformatics Analysis

#### 3.4.1. Prediction of Aerobic Exercise-Depression Related Target Genes and Construction of PPI Network

**Figure 7:**
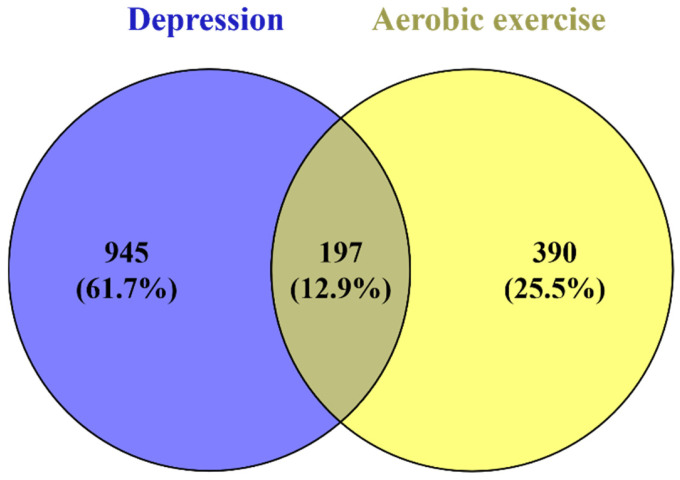
VENN plot of aerobic exercise target and depression target.

The obtained targets related to “Depression” and “Aerobic exercise” were screened using twice the median relevance score as the criterion. A total of 1142 targets related to “Depression” and 587 targets related to “Aerobic exercise” were obtained respectively. These related targets were mapped using VENNY 2.1, and the overlapping targets were extracted, resulting in 197 overlapping targets (Figure 7).

#### 3.4.2. PPI Network Construction

**Figure 8:**
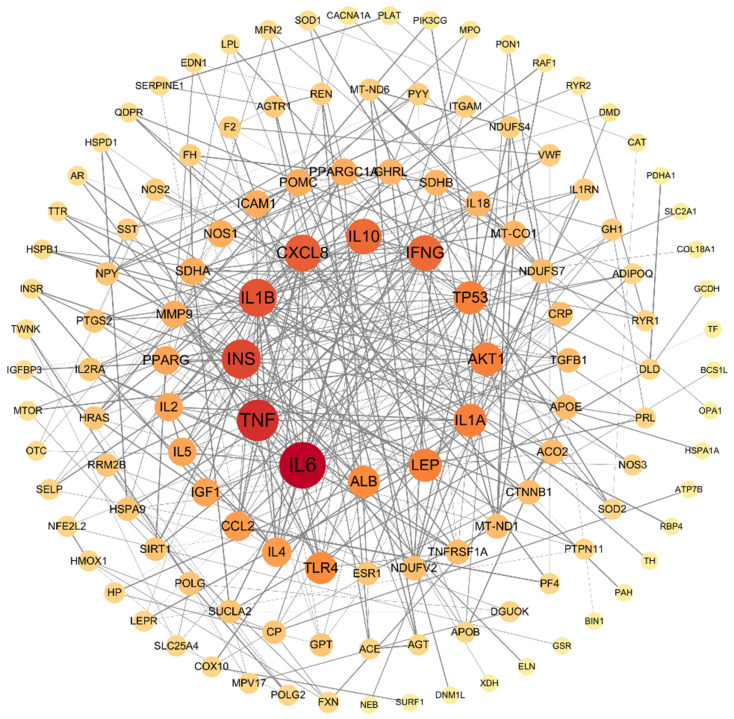
PPI network for aerobic exercise-depression targets.

To explore the interaction relationships among potential targets, the screened overlapping targets were imported into the String 12.0 platform, and a PPI network was constructed using Cytoscape. As shown in Figure 8, this network consists of 128 nodes and 366 edges. Topological structure analysis was performed using the MCODE plugin in Cytoscape 3.10.3. The results indicated that IL6, TNF, INS, IL1B, and other genes are predicted to be potential targets for the antidepressant effect of aerobic exercise.

#### 3.4.3. GO Enrichment Analysis and KEGG Pathway Analysis

**Figure 9:**
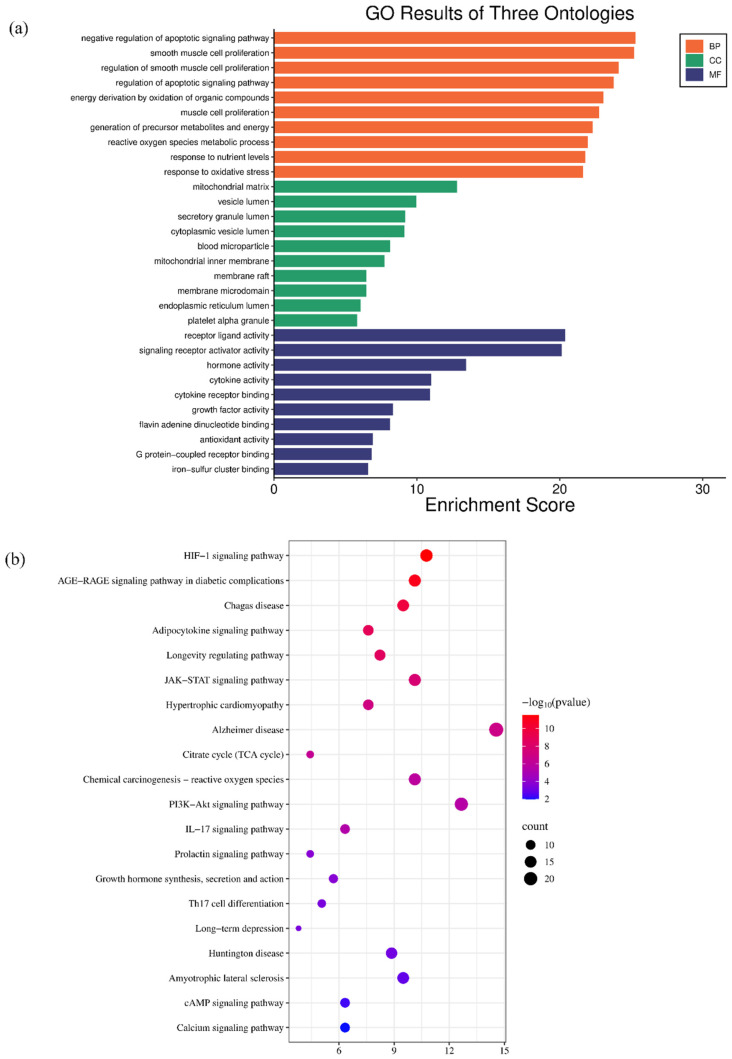
Enrichment analysis of potential targets for aerobic exercise-depression. (**a**) GO enrichment analysis; (**b**) KEGG analysis.

The DAVID database was used for enrichment analysis. The top 10 GO terms in biological process (BP), cellular component (CC), and molecular function (MF) categories were selected for visualization (Figure 9a). For the potential targets of aerobic exercise in depression intervention: The involved biological processes mainly included negative regulation of apoptotic signaling pathway, smooth muscle cell proliferation, muscle cell proliferation, and response of precursor metabolites and energy production to oxidative stress. The related cellular components mainly covered mitochondrial matrix, vesicle lumen, blood microparticle, and inner mitochondrial membrane. The associated molecular functions mainly involved receptor ligand activity, signal receptor activator activity, cytokine receptor binding, and growth factor activity. The top 20 KEGG pathways were selected for visualization (Figure 9b). The main involved pathways included Hypoxia-inducible factor-1 (HIF-1) signaling pathway, advanced glycation end-products-receptor for advanced glycation end-products (AGE-RAGE) signaling pathway, Chagas disease, and adipocytokine signaling pathway (Figure 9).

#### 3.4.4. Target-Pathway Network Construction and Core Targets Screening

**Figure 10:**
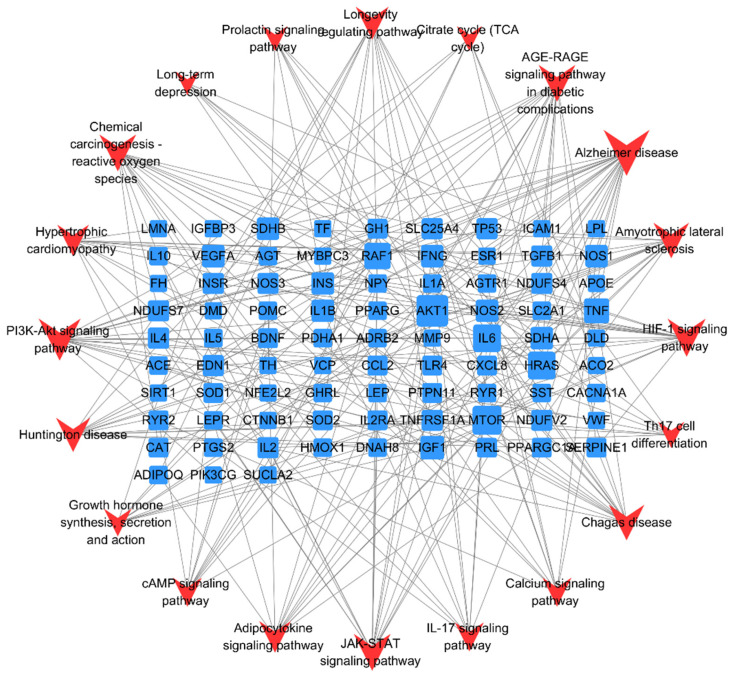
Target-pathway diagram of aerobic exercise intervention for depression.

A “target-pathway” network diagram was constructed using CytoScape 3.7.2. Core functional targets were identified by analyzing the topological parameters of the target network for aerobic exercise intervention in depression. Aerobic exercise intervened in depression through multiple targets, and core targets were determined based on Degree values. The top five targets ranked by Degree value were protein kinase B (AKT1), Mammalian Target of Rapamycin (MTOR), interleukin-6 (IL6), RAF Proto-Oncogene Serine/Threonine Kinase 1 (RAF1), and Tumor Necrosis Factor (TNF) (see Table 6). These targets were predicted as the core targets of aerobic exercise in intervening depression, while the remaining targets were considered relatively important ones (Figure 10, Table 6).

## 4. Discussion

The CUMS model is a classic animal model in the field of depression research. Sustained stimulation from chronic unpredictable mild stress leads rats to exhibit core depressive symptoms such as reduced reward response and anhedonia, making it considered the animal model that most closely mimics depressive behaviors in humans [17]. The positive effects of aerobic exercise on depression have been confirmed by previous studies. In this research, behavioral tests on CUMS rats showed that 4 weeks of aerobic exercise significantly improved the rats’ anhedonia, as well as their spontaneous activity and exploratory behavior. The occurrence of these depressive-like behaviors was accompanied by abnormal changes in some blood biochemical indicators and urine metabolites in CUMS rats. Aerobic exercise’s ability to partially reverse these abnormal metabolic changes may be one of the mechanisms by which it improves depressive-like behaviors.

### 4.1. Four Weeks of Aerobic Exercise Enhances Anti-Fatigue Ability in CUMS Rats

Psychomotor retardation is a core symptom of depression, along with low mood and loss of interest or pleasure [18]. In human studies, blood testosterone and cortisol levels are often used to reflect the body’s anabolic and catabolic metabolism. A decrease in blood testosterone concentration and/or an increase in cortisol concentration is regarded as an important indicator of reduced exercise capacity and increased fatigue [19]. Both cortisol and corticosterone are glucocorticoids secreted by the adrenal cortex, but they differ in their secretion sites, physiological functions, and species distribution. Cortisol is the main glucocorticoid in humans and primates, involved in stress response, metabolic regulation, and immune suppression. In contrast, corticosterone is dominant in rodents; it is secreted only in small amounts in humans and plays a relatively minor role [20]. In animal studies, depression models are often induced by corticosterone injection because corticosterone stimulation may damage intestinal barrier integrity, trigger substantial secretion of pro-inflammatory factors, and cause hyperactivity of the hypothalamic-pituitary-adrenal (HPA) axis, ultimately leading to depression [21].

Ammonia is a product of protein metabolism. Under normal conditions, ammonia from the intestines enters the liver and is cleared there. However, in a depressed state, liver atrophy reduces the ability to clear ammonia [22], leading to an increase in blood ammonia concentration. When high concentrations of blood ammonia enter the central nervous system, they not only disrupt neurotransmission but also impair intracellular homeostasis, exacerbating depressive symptoms [23]. This study found that serum corticosterone and blood ammonia levels were significantly elevated in CUMS rats, and that these levels decreased significantly following aerobic exercise. This suggests that four weeks of aerobic exercise enhanced the anti-fatigue ability of CUMS rats.

Metabolomics is a powerful technique for elucidating the pathological mechanisms of diseases. It can screen for disease-related biomarkers at the small-molecule metabolite level through the analysis of biological samples. Previously, our research group analyzed different biological samples from CUMS depression model rats using various metabolomics techniques and conducted preliminary studies on the metabolic regulatory mechanisms by which exercise intervention improves depression [24]. In this study, we combined LC-MS metabolomics (which offers higher sensitivity and more accurate detection) with ^1^H-NMR metabolomics (which detects a broader range of substances) to comprehensively and deeply illustrate the regulatory effect of aerobic exercise on the urine metabolite profile of CUMS rats. Additionally, using bioinformatics techniques, we predicted the core protein targets and signaling pathways involved in aerobic exercise intervention for depression.

### 4.2. Four Weeks of Aerobic Exercise Significantly Regulates Urine Metabolic Remodeling in CUMS Rats

In this study, we employed LC-MS and ^1^H-NMR metabolomics techniques to investigate changes in the urine metabolic profile of CUMS rats and the effects of a four-week aerobic exercise intervention. In the present study, the overlap of metabolites detected by LC-MS and ^1^H-NMR was relatively low, with glutamine being the only common metabolite identified by both techniques. This phenomenon could be attributed to the inherent differences in their detection characteristics: ^1^H-NMR is more suitable for the detection of structurally stable metabolites with high abundance [25], whereas LC-MS exhibits higher sensitivity toward polar small-molecule metabolites [26].Combining these two metabolomic approaches enhanced the credibility and comprehensiveness of our findings and provided a novel methodology for understanding the metabolic characteristics of depression and the mechanisms underlying exercise intervention.

Both LC-MS and ^1^H-NMR analyses identified three metabolic pathways associated with differential urine metabolites in CUMS rats: “arginine and proline metabolism,” “glycine, serine and threonine metabolism,” and “alanine, aspartate and glutamate metabolism.” This consistently indicates that amino acid metabolism disruption is a key metabolic feature in CUMS rats. Amino acids are not only the building blocks of proteins but also play crucial roles in cell signaling, molecular regulation, and glucose and lipid metabolism. Their systemic dysregulation is likely to affect neurotransmitter balance, energy homeostasis, and epigenetic processes such as methylation, thereby contributing to the pathophysiology of depression [27].

Methionine (Met) serves as an important component in the methionine cycle. Studies show that under the action of methionine adenosyltransferase, Met is converted to S-adenosylmethionine (SAMe), a key intermediate that supplies methyl groups and participates in the synthesis of various physiologically active substances, including epinephrine, creatine, and dopamine. Aberrant SAMe levels or function can impair neurotransmitter synthesis and methylation, leading to depression [28]. Ullah et al. [29] found that daily supplementation with SAMe and probiotic strains for three months effectively alleviated depressive symptoms. In this study, urine methionine levels were significantly reduced in CUMS rats and were significantly increased after aerobic exercise, suggesting that exercise may exert antidepressant effects by modulating cysteine and methionine metabolism.

Previous studies have confirmed that hippocampal neuronal damage occurs in depressed rats [30]. Astrocytes convert glutamate into glutamine, which is released into the extracellular space, taken up by neurons, and reconverted to glutamate [31]. Glutamate is an excitatory neurotransmitter, and abnormally elevated levels can cause neuronal damage and trigger depression [32]. In the present study, abnormal glutamine levels in CUMS rats were detected by both metabolomic techniques, indicating that the glutamate-glutamine cycle in the body may be impaired, which in turn induces depressive-like behaviors in the rats. Notably, abnormal glutamine levels may also be associated with liver metabolic dysfunction (CUMS can induce liver tissue damage), and the differences in regulatory mechanisms between the central nervous system and peripheral tissues require further verification with brain and liver tissue samples.

Tyramine is a biogenic amine widely present in living cells and plays important physiological roles, such as promoting growth and enhancing metabolic activity. However, excessive accumulation can produce toxic effects, leading to conditions such as hypertension and headaches, and may contribute to other pathologies [33]. Tyrosine, an essential aromatic amino acid, serves as a precursor for neurotransmitters including epinephrine, norepinephrine, and dopamine. Tyrosine is decarboxylated by tyrosine decarboxylase to form tyramine. Tyrosine supplementation under stress can prevent norepinephrine depletion in depressed patients and alleviate depressive symptoms [34]. In this study, urine tyramine levels were significantly increased in CUMS rats, indicating disrupted tyrosine metabolism. After aerobic exercise, tyramine levels significantly decreased, suggesting that modulation of tyrosine metabolism may be one mechanism through which exercise exerts antidepressant effects.

The present study demonstrated that aerobic exercise significantly reversed the levels of five differential metabolites (caprolactam, cholic acid, tyramine, indoxyl sulfate, and methylthioadenosine) in the urine of CUMS rats, indicating a trend toward metabolic recovery. However, the number of reversed metabolites was relatively limited, which may be attributed to three main factors. First, the acute high-intensity interval training (HIIT) protocol used in this study exerts a rapid metabolic stress response but lacks the long-term regulatory effect required to rectify multiple disordered metabolic pathways. Second, individual differences in the CUMS rat model lead to heterogeneous damage to the neuro-endocrine-metabolic network, with some rats exhibiting irreversible metabolic disorders. Third, the detection limitations of the combined LC-MS and NMR techniques, coupled with the low abundance and short half-lives of certain depression-related metabolites, may have prevented the identification of more reversed metabolites. [35]. Changes in the aforementioned metabolites reflect that the gut microbiota may have undergone remodeling [36], and future studies are expected to perform 16S rRNA sequencing of the gut microbiota for direct verification [37].

It should be emphasized that only 6 differential metabolites showed significant reversal after exercise intervention, which may be related to multiple factors. In addition to the previously mentioned short intervention duration and individual differences in the CUMS rat model, the exercise intensity may not have reached the optimal response threshold for some rats, or the exercise frequency may not have fully covered the repair cycle of metabolic pathways, which may also lead to significant reversal of only 6 differential metabolites.

Our research group has previously analyzed the metabolic profile of hippocampal tissue in CUMS rats using LC-MS technology, identifying 16 depression-related differential metabolites. Among these, 8 metabolites (including glutamine, glutamate, L-phenylalanine, tyrosine, and hypoxanthine) were significantly reversed following aerobic exercise intervention. Additionally, 5 core regulatory pathways were clarified, such as the biosynthesis of phenylalanine, tyrosine, and tryptophan, as well as glutamine and glutamate metabolism [38].

Comparative analysis revealed that core pathways including “alanine, aspartate and glutamate metabolism” and “phenylalanine metabolism” were consistently regulated by aerobic exercise in both urine and hippocampal tissues. Moreover, key metabolites such as L-phenylalanine, tyrosine, glutamine, and glutamate exhibited highly consistent regulatory trends in the central nervous system and peripheral tissues following exercise. Meanwhile, the regulation of N-acetylaspartate (NAA)—a key marker of central hippocampal energy metabolism—and 3-hydroxyvaleric acid—a peripheral urinary fatty acid metabolite—by aerobic exercise also reflected the systemic characteristics of improved energy metabolism.

This dual overlap of pathways and metabolites establishes a mechanistic link between peripheral urinary metabolic changes and central hippocampal neurochemical alterations, bridging the gap between systemic metabolism and brain function in the context of exercise intervention for depression. It significantly enhances the comprehensiveness and rationality of interpreting the antidepressant mechanism of aerobic exercise.

### 4.3. Elucidating the Potential Mechanism of Aerobic Exercise in Alleviating Depression Using Bioinformatics

Aerobic exercise exerts antidepressant effects by modulating multiple signaling pathways in the body. Based on an analysis of the topological parameters of the target network, AKT1, mTOR, IL-6, RAF1, and TNF were predicted to play central roles. These targets are predicted based on bioinformatics analysis of overlapping genes, and their direct regulatory effects on depressive-like behaviors require in vitro (e.g., cell culture) and in vivo (e.g., gene knockout) validation. Within the central nervous system, reduced AKT activity is closely linked to the development of anxiety and depression. AKT activation depends on the PI3K signaling pathway, with mTOR serving as a key downstream effector. Studies indicate that decreased mTOR expression in the infralimbic prefrontal cortex can induce depressive-like behaviors in mice [39], whereas both aerobic and resistance exercise upregulate phosphorylated mTOR in relevant brain regions, contributing to antidepressant effects [40].

As a pleiotropic cytokine, IL-6 exhibits elevated pro-inflammatory activity during the pathogenesis of depression, which is positively correlated with symptom severity [41]. Aerobic exercise can significantly reduce elevated IL-6 levels in both the peripheral and central nervous systems and promote BDNF release, thereby conferring a neuroprotective effect [42]. RAF1 is a key upstream regulator of the MAPK/ERK signaling pathway, playing a vital role in cell survival, proliferation, and the maintenance of neural plasticity. In the brains of individuals with depression, impaired MAPK/ERK signal transduction leads to reduced synaptic plasticity and weakened neuronal connectivity, thereby exacerbating emotional dysregulation [43]. Although direct evidence linking RAF1 to improved depressive-like behaviors via MAPK/ERK regulation is currently lacking, its hub position within the signaling network suggests that RAF1 may be an important potential target for non-pharmacological interventions (such as exercise). Modulating RAF1 could influence neural plasticity and energy metabolism, thereby contributing to antidepressant effects.

TNF-α is a major pro-inflammatory cytokine directly involved in the neuroinflammatory processes underlying depression. Clinical studies report elevated TNF-α levels in depressed patients [44], and animal experiments demonstrate that aerobic exercise effectively reduces hippocampal TNF-α in depression model rats [45]. The above results suggest that the antidepressant effect of aerobic exercise may exhibit a multi-target characteristic, where these targets are interconnected and are predicted to jointly act on signaling pathways to exert antidepressant effects.

The metabolic pathways and core targets identified in this study only reflect one of the potential mechanisms of aerobic exercise against depression. For example, the activation of the PI3K-AKT pathway may also be related to exercise-induced improvement in insulin sensitivity, rather than direct regulation of depression-related neural functions; the downregulation of IL-6 may not only alleviate neuroinflammation but also affect the metabolic adaptation of skeletal muscle. Future studies need to clarify the specific role of these pathways and targets in exercise-induced antidepressant effects by blocking specific pathways or targets.

Integrating metabolomics with bioinformatics, this study elucidated how a four-week aerobic exercise intervention regulates urinary metabolic remodeling in CUMS rats. It provides further mechanistic evidence supporting the “exercise-metabolism-depression” link and offers new experimental validation for the metabolic dysregulation hypothesis of depression. Furthermore, the study demonstrates that four weeks of aerobic exercise effectively ameliorates metabolic disturbances and enhances anti-fatigue capacity in a depression model, offering a specific duration reference for designing clinical exercise interventions. Finally, the metabolic analysis of urine samples points to a promising non-invasive approach for early depression screening and the assessment of exercise intervention efficacy in future research.

## 5. Conclusions

**Figure 11:**
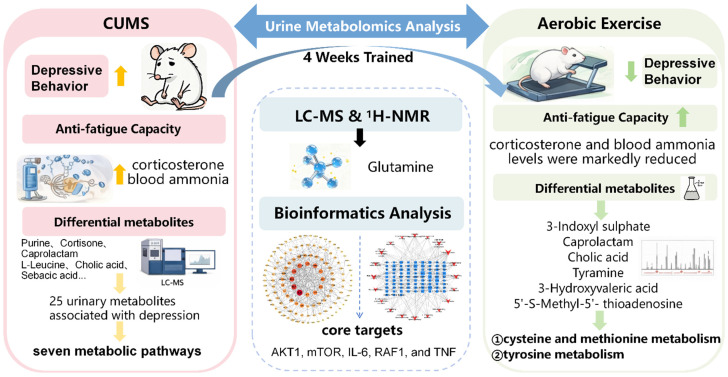
Aerobic Exercise Mechanism Diagram for Alleviating Depression.

A four-week regimen of aerobic exercise significantly improved depressive-like behaviors and enhanced anti-fatigue capacity in CUMS rats. This exercise regimen promoted urinary metabolic remodeling, primarily associated with the modulation of amino acid metabolism. Furthermore, its antidepressant effect likely potentially involves the regulation of core tissue targets—including AKT1, mTOR, IL-6, RAF1, and TNF—thereby influencing key pathways such as PI3K-AKT, MAPK/ERK, and neuroinflammatory signaling (Figure 11).

## 6. Limitations

This study has several limitations. Core targets and pathways identified by metabolomics-bioinformatics analysis lack experimental validation, with follow-up molecular biology validation and targeted metabolomics assays planned. Only male rats were used, limiting conclusion generalization to females due to depression’s sexual dimorphism, and future studies will include female subjects. The single fixed exercise protocol prevents extrapolation to other modalities, necessitating systematic comparison of diverse exercise parameters. This study only establishes a correlation, not causation, between exercise-induced amino acid metabolic remodeling and antidepressant effects, to be confirmed via inhibitor-based experiments. Furthermore, quantitative multi-omics integration of metabolomics and genomics is planned for future studies.

## Figures and Tables

**Table 1:** Specific Aerobic Exercise Protocol.

Stage Type	Intervention Duration/Frequency	Treadmill Speed (m/min)& Time Arrangement
Treadmill Familiarization	3 days prior to the formal exercise; 15 min per day	3 m/min (5 min) → 5 m/min (5 min) → 8 m/min (5 min)
Formal Treadmill Exercise	5 times per week; 30 min per session	3 m/min (5 min) → 5 m/min (5 min) → 8 m/min (20 min)

**Table 2:** Biochemical indicators of fatigue in all groups of rats (x ± s, n = 8).

Group	CORT/(ng/mL)	T/(ng/mL)	LD/(mmol/L)	Hb/(g/L)	NH_3_/(μmol/L)
C	293.76 ± 24.15	4.93 ± 0.62	38.28 ± 2.36	136.22 ± 10.26	61.22 ± 2.68
E	306.26 ± 25.82 ^#^	4.68 ± 0.73	37.92 ± 2.86	129.88 ± 9.68	60.93 ± 2.57 ^##^
D	417.42 ± 27.88 **	4.19 ± 0.69	35.13 ± 2.75	131.73 ± 14.45	69.32 ± 5.42 **
DE	321.38 ± 23.54 ^##^	4.16 ± 0.65	36.53 ± 2.28	123.85 ± 10.92	63.87 ± 4.33 ^##^

** *p* < 0.01 versus Group C; ^#^ *p* < 0.05, ^##^ *p* < 0.01 versus Group D.

**Table 3:** Differential metabolites between groups C and D and between groups D and DE.

No.	Metabolites	Formula	TR (min)	C/D	D/DE
1	Phenylacetylglycine	C_10_H_11_NO_3_	11.602	↓ **	—
2	3-Indoxyl sulphate	C_8_H_7_NO_4_S	9.748	↓ **	↑ ^##^
3	7-Methylguanine	C_6_H_7_N_5_O	2.17	↓ **	—
4	Mesaconic acid	C_5_H_6_O_4_	1.967	↓ **	—
5	Indole-3-acetic acid	C_10_H_9_NO_2_	15.178	↓ **	—
6	Purine	C_5_H_4_N_4_	1.09	↓ **	—
7	Cortisone	C_21_H_28_O_5_	19.164	↓ **	—
8	Caprolactam	C_6_H_11_NO	9.357	↑ **	↓ ^#^
9	L-Leucine	C_6_H_13_NO_2_	12.194	↓ *	—
10	Cholic acid	C_24_H_40_O_5_	19.958	↑ **	↓ ^##^
11	Sebacic acid	C_10_H_18_O_4_	12.329	↓ *	—
12	4-Guanidinobutyric acid	C_5_H_11_N_3_O_2_	1.776	↓ **	—
13	Guanidineacetic acid	C_3_H_7_N_3_O_2_	1.022	↓ **	—
14	5-Methylcytosine	C_5_H_7_N_3_O	3.365	↓ **	—
15	N-Acetyl-DL-glutamic acid	C_7_H_11_NO_5_	2.59	↓ **	—
16	L-(-)-Methionine	C_5_H_11_NO_2_S	1.876	↓ *	—
17	N-Acetyl-L-methionine	C_7_H_13_NO_3_S	9.381	↓ **	—
18	Nicotinic acid	C_6_H_5_NO_2_	1.268	↓ **	—
19	N-Acetylaspartic acid	C_6_H_9_NO_5_	1.819	↓ **	—
20	3-Hydroxybutyric acid	C_4_H_8_O_3_	3.236	↓ **	—
21	Tyramine	C_8_H_11_NO	1.086	↑ **	↓ ^##^
22	3-Hydroxyvaleric acid	C_5_H_10_O_3_	5.686	↓ **	↑ ^##^
23	Hypoxanthine	C_5_H_4_N_4_O	5.462	↓ **	—
24	5′-S-Methyl-5′-thioadenosine	C_11_H_15_N_5_O_3_S	9.332	↓ *	↑ ^#^
25	Kynurenic acid	C_10_H_7_NO_3_	8.942	↑ **	—

Comparison with group C: * *p* < 0.05, ** *p* < 0.01; comparison with group D: ^#^ *p* < 0.05, ^##^ *p* < 0.01. ↑ indicates an increase in the content of the metabolite, ↓ indicates a decrease in the content of the metabolite.

**Table 4:** LC-MS and ^1^H-NMR study of urinary metabolomics of depression-related metabolites in CUMS rats.

Technical	Depression-Related Metabolites	Depression-Related Metabolic Pathways
^1^H-NMR	acetic acid, glycoprotein, glutamine, dimethylamine, methylguanidine, trimethylamine, creatine, butanediamine, trimethylamine oxide, acetoacetic acid, malic acid, fenugreek, marinic acid, acetone, pyruvic acid, a-ketoglutaric acid, citric acid, malonic acid, glycine, creatinine, guanine	**alanine, aspartate and glutamate metabolism,** TCA cycle, glycolysis or gluconeogenesis, butyrate metabolism, pyruvate metabolism, **arginine and proline metabolism,** **glycine, serine and threonine metabolism,** ketone body synthesis and degradation
LC-MS	phenylacetylglycine, indolephenol sulfate, 7-methylguanine, methylfumaric acid, indoleacetic acid, purines, adrenocorticotropic ketone, caprolactam, L-leucine, cholic acid, sebacic acid, 4-guanidinobutanoic acid, guanidinic acid, 5-methylcytosine, N-acetyl-DL-glutamate, L-methionine, N-acetyl-L-methionine, nicotinamide, N-acetyl-L-aspartate, 3-hydroxybutyrate, tyrosine, 3-hydroxyvaleric acid, 3-hydroxypentanoic acid. hydroxybutyric acid, tyramine, 3-hydroxyvaleric acid, hypoxanthine, methylthioadenosine, kynurenic acid	cysteine and methionine metabolism, **alanine, aspartate and glutamate metabolism,** **arginine and proline metabolism,** **glycine, serine and threonine metabolism,** tyrosine metabolism, purine metabolism, steroid hormone biosynthesis

The bolded portion represents the depression-related metabolic pathways shared by both metabolomics techniques.

**Table 5:** Analysis of aerobic exercise modulates changes in urinary depression-related differential metabolites in CUMS rats based on LC-MS and ^1^H-NMR metabolomics techniques.

Technical	Exercise Regulated Metabolites	Exercise Regulated Metabolic Pathways
^1^H-NMR	glutamine, acetone, pyruvate, creatine, cucurbitacine	alanine, aspartate and glutamate metabolism, TCA cycle, glycolysis or gluconeogenesis, butyric acid metabolism, pyruvic acid metabolism
LC-MS	indolephenol sulfate, caprolactam, cholic acid, tyramine, hydroxyvaleric acid, 3-methylthioadenosine	cysteine and methionine metabolism, tyrosine metabolism

**Table 6:** Characteristic parameters of the node nodes of aerobic exercise intervention depression.

Name	Degree	Betweenness Centrality	Closeness Centrality
AKT1	12	0.12128	0.45575
MTOR	10	0.07349	0.41532
IL6	9	0.05853	0.40234
RAF1	8	0.04878	0.39615
TNF	7	0.05168	0.39312

## Data Availability

The original contributions presented in this study are included in the article. Further inquiries can be directed to the corresponding authors.
